# Case Report: Treatment of systemic mastocytosis with sunitinib

**DOI:** 10.12688/f1000research.13343.1

**Published:** 2017-12-28

**Authors:** Gerhard J. Molderings, Lawrence B. Afrin, Hans-Jörg Hertfelder, Stefan Brettner

**Affiliations:** 1 Institute of Human Genetics, University Hospital Bonn, Bonn, D-53127, Germany; 2 Armonk Integrative Medicine, Armonk, NY, 10504, USA; 3 Institute of Experimental Haematology & Transfusion Medicine, University Hospital of Bonn, Bonn, D-53127, Germany; 4 Department of Oncology, Hematology and Palliative Care, Kreiskrankenhaus Waldbröl, Waldbröl, D-51545, Germany

**Keywords:** mast cell, systemic mast cell activation disease, systemic mastocytosis, sunitinib, tyrosine kinase inhibitors

## Abstract

Mast cell activation disease typically presents as chronic multisystem polymorbidity of generally inflammatory ± allergic theme.  Presently, treatment of the rare, cytoproliferative variant systemic mastocytosis employs empirically selected therapies to impede mast cell mediator production and action and, when necessary, inhibition of proliferation. Some tyrosine kinase inhibitors (TKIs) have been used successfully in uncommon cases of systemic mastocytosis not bearing that disease’s usual imatinib-resistant KIT ^D816V^ mutation. Recently, sunitinib, a multi-targeted TKI, had been successful in a case of systemic mast cell activation syndrome. In addition, most allergy is principally a mast cell activation phenomenon, and sunitinib has been shown helpful in controlling a murine model of oral allergy syndrome. Here, we present the first use of sunitinib in systemic mastocytosis.

## Introduction

Systemic mastocytosis (SM) represents the rare variant (prevalence in Europeans ranges between 0.3 and 13:100,000) ^reviewed in 1^ of systemic mast cell activation disease (MCAD; Table S1). MCAD comprises a heterogeneous group of multifactorial, polygenic disorders characterized by aberrant release of variable subsets of mast cell (MC) mediators together with accumulation of either morphologically altered and immunohistochemically identifiable mutated MCs due to MC proliferation (SM and mast cell leukemia) or morphologically ordinary MCs due to decreased apoptosis (well-differentiated SM and systemic mast cell activation syndrome) ^for details, see 2^.

Due to its genetic and epigenetic roots, MCAD generally is regarded as incurable. Recent mutational studies ^reviewed in 3^ revealed an individual pattern of genetic and epigenetic alterations for each patient, which may affect the receptor expression, and their intracellular signal transduction pathways. Hence, mediator formation and release as well as inhibition of apoptosis, and/or increase in proliferation, are determined by individual genetic and epigenetic conditions requiring a highly personalized therapy for the disease. In formal studies in SM patients, although some tyrosine kinase inhibitors (TKIs) reduced MC burden, as reflected by histological normalization in bone marrow and improved laboratory surrogate markers (e.g., blood tryptase level), at best only partial improvement of mediator-related symptoms was achieved ^4– 7^. Distinction in pathways in the MC that promote MC proliferation vs. mediator production/release may explain why TKIs reduce MC burdens and MC-driven symptoms to different degrees ^4– 7^. However, in some case reports, TKIs have been significantly effective at relieving symptoms. Thus, in spite of a potential for serious adverse effects of these drugs, therapeutic trials may be justified in individual cases at various stages in their courses. Possibly due to the causative mutations in multiple genes leading to simultaneous activation of multiple intracellular pathways, multi-targeted TKIs such as midostaurin and sunitinib may be more effective than drugs which selectively downregulate only one intracellular pathway. Sunitinib has been shown to be helpful in a murine model of oral allergy syndrome, a disease likely rooted in aberrant MC activation ^8^.

## Case presentation

A 58-year-old man presented to our *Interdisciplinary Multicenter Research Group for Systemic Mast Cell Diseases* for therapy of his progressive aggressive systemic mastocytosis (ASM, Table S1 and Table S2), diagnosed according to the WHO criteria ( Table S3), which remained significantly symptomatic despite the use of drugs administered to reduce MC activation ^reviewed in 9^ (prednisone, rupatadine, ranitidine, ascorbic acid, ketotifen, montelukast, omalizumab) and drugs administered to reduce mediator-related symptoms (omeprazole, candesartan, risedronic acid, clonidine, cholestyramine, tranexamic acid, metamizole). A previous therapeutic trial with methotrexate was unhelpful. As typical in SM, his full history is complex, and symptoms reported, and key laboratory results found, are summarized in Table 1. The only minimal elevation in tryptase in this case of not just SM but in fact *aggressive* SM illustrates the heterogeneity of SM/ASM.

**Table 1.** Symptoms and Key Laboratory Results.

Symptoms	Key Laboratory Results (normal range)
Fatigue; malaise; asthenia; feeling cold much of the time; headache; word finding difficulties; “brain fog”; attention deficit disorder; sleep disruptions; body shivering; restless-leg-like symptoms; short-term myoclonus; high startle response; central coordination disorder; constant bilateral tinnitus; irritated eyes; nasal irritation and copious coryza; wheezing; irritated throat during flares; dyspnea; dry cough; desire to clear one's throat; formation of a viscous mucus; chest discomfort/heaviness; palpitations; hot flash; arterial hypertension; intermittent tachycardic sinus arrhythmias; secondary Raynaud´s syndrome; “easy” bruising/bleeding; nausea; diarrhea; marked abdominal bloating; recurrent splenomegaly; hypercholesterolemia; heartburn; diffuse edema with weight gain for several days; diffusely migratory paresthesias and pain; rheumatoid arthritis-like symptoms; flushes; itching without rashes; mouth ulcers; intolerance of a large number of foods, gluten, lactose, and chemical substances; gastritis; colitis; osteoporosis; waxing/waning bilateral sore throat; chronic kidney failure grade 1; dermatographism; longitudinal ridging in all nails; mood disturbances; recurrent impaired vision	Mast cell clusters (>15 MCs) in gastro-intestinal biopsies; 14% were stained CD25-positive; somatic KIT ^D816V^ mutation and alterations in KIT outside codon 816; Serum tryptase: 15.8 µg/L (normal range <11.5 µg/L); Recurrent spontaneous fractures; Recurrent hepatic dysfunction; Plasma heparin level progressively increasing since the time of diagnosis; Clotting factor VIII increased; Trigger-induced increase of leukotrienes in blood; Severe IgA-deficiency in blood and saliva: Waxing/waning low-titer autoantibodies without corresponding symptoms in the respective organs; Decrease of thrombocytes from 197,000/µL to 114,000/µL (normal range 150,000 – 350,000/µL) and of the amount of total protein in blood to 5.5 g/dL (normal range 6.60 – 8.70 g/dL) Increase in uric acid from 5.6 to 7.2 mg/dL (normal 3.4 – 7.0 mg/dL) Mutation analysis of genomic DNA of leukocytes from peripheral blood by next generation sequencing: germline mutations in coding sequences: TET2 ^I1762V^ (heterozygously) IL13 ^Q144R^ (homozygously) TP53 ^P72R^ (homozygously) SETBP1 ^A222T, T228Sfs*8^ (heterozygously)

Since recently sunitinib had been used successfully in a case of systemic mast cell activation syndrome ^10^ ( Table S1), we decided for an off-label trial with sunitinib. Sunitinib is a multi-targeted TKI (up to 313 potential kinase targets) ^reviewed in 9^ which, in addition to KIT, also binds to PDGFR-α, PDGFR-β, VEGFR1, VEGFR2, VEGFR3, FLT3, CSF-1R, and RET, some of which are also expressed in MCs. The patient gave written informed consent to participate in the off-label therapeutic trial with sunitinib, which is approved to treat imatinib-resistant, largely KIT-mutation-driven gastrointestinal stromal tumor and other applications, but not yet systemic mastocytosis ^reviewed in 11^. For such a therapeutic trial, ethical approval is not necessary in Germany ^a^. There was no contra-indication for use of sunitinib in the patient, in particular no sign of abdominal aortic aneurysm. We now report the first use of sunitinib in systemic mastocytosis.

In a first attempt, the patient took 12.5 mg sunitinib once daily for 24 days. After just three days, the abnormal bleeding (e.g. intense gum bleeding) he had due to increased fibrinolysis, which is a typical symptom in MCAD ^12, 13^, ceased. The multiple subcutaneous fibrotic nodules that had developed all over his body during his many years of SM became tender and movable in the skin. Although no other symptoms were improved and sunitinib did not prevent flares of the disease, the patient felt better subjectively, in particular with less fatigue. However, in parallel the body hair became depigmented (white) and there was a decrease both in the number of thrombocytes and in the amount of total protein in blood, whereas uric acid in the blood increased inducing gout ( Table 1). At that point treatment with sunitinib was discontinued.

When the altered blood parameters had returned to the normal ranges 33 days later, the patient asked for a second treatment try. During that time, the positive effects of the first period of sunitinib use had vanished. This second treatment, again with 12.5 mg sunitinib once daily, lasted only 8 days, during which the same positive and negative effects occurred as during the first intake, so the second therapeutic trial had to be terminated, too. It is possible that an even lower dose of the drug (e.g., 6.25 mg, which would pose some (surmountable) challenges, since the lowest capsule dose available is 12.5 mg), might have provided equal benefit with less toxicity, especially in view of the many case reports now of imatinib-responsive SM showing remarkable sensitivity to doses of that drug far below what seems necessary in most other oncologic applications of the drug. Cyclic rather than continuous dosing of sunitinib was shown to be the optimal approach in the approved indications for that drug. It may be the same, albeit using a cycling pattern different than for oncologic/anti-neoplastic applications, might prove true with use of sunitinib in SM.

## Conclusions

In conclusion, this ASM partially responded to sunitinib. However, in this patient toxicities outweighed limited benefits. A similar benefit-risk assessment of sunitinib was observed in a second patient with ASM (unpublished case; GJM). However, our findings do not rule out that therapeutic trials with sunitinib may be reasonable in other SM patients whose disease is refractory to other TKIs.

## Consent

Written informed consent for publication of his clinical details was obtained from the patient.

## Notes

^a^ In Germany, individual therapeutic trials are not regulated by law. They are not notifiable, and there is no register for it. It is recognized that the freedom of medical treatment also includes the individual therapeutic attempt. The freedom of medical treatment has a legal basis in sub-constitutional laws and in regulations of the German medical association. In the end it is based on the freedom to pursue an occupation guaranteed by article 12 subparagraph 1 of the German constitution.

## Supplementary material

**Table S1.** Classification of mastocytosis.

**Table S2.** C-Findings = Indication of impaired organ function due to MC infiltration defining a SM as an aggressive SM.

**Table S3.** WHO criteria defining *Systemic Mastocytosis*.
