# Supplementary material for: Case Report: Treatment of systemic mastocytosis with sunitinib
Source: F1000Res. 2017 Dec 28;6:2182. [Version 1] doi: 10.12688/f1000research.13343.1 (PMC5946163; doi:10.12688/f1000research.13343.1)
Supplement: Supplementary file 1 [file f1000research-6-14480-s0000.tgz › c8aab504-c7f1-495a-9841-3a514ddb39a8.pdf]

Supplemental Table S1: Current classification of primary mastocytosis

<table>
<tr><th colspan="4">Mastocytosis</th></tr>
<tr><th colspan="2">Systemic mast cell activation disease (MCAD)</th><th rowspan="2">Cutaneous mastocytoses (CM)</th><th rowspan="2">Mast cell sarcoma</th></tr>
<tr><th>Systemic Mastocytosis (SM)</th><th>Mast Cell Activation Syndrome (MCAS)</th></tr>
<tr><td>• indolent SM
• well-differentiated indolent SM
• smoldering SM
• aggressive SM
• SM with an associated leukemia
• mast cell leukemia</td><td>• with hypertryptasemia
• without hypertryptas-emia</td><td>• maculopapular mastocytosis = urticaria pigmentosa (UP)
• diffuse CM
• solitary cutaneous mastocytoma
• telangiectasia macularis eruptiva perstans</td><td></td></tr>
</table>

Based on:
Vysniauskaite M et al. Determination of plasma heparin level improves identification of systemic mast cell activation disease. PLoS One 2015;10:e0124912.
Afrin LB et al. Often seen, rarely recognized: mast cell activation disease--a guide to diagnosis and therapeutic options. Ann Med 2016;48190-201.
Arber A et al. The 2016 revision to the World Health Organization (WHO) classification of myeloid neoplasms and acute leukemia. Blood 2016;127:2391-405.
