# Supplementary material for: Case Report: Treatment of systemic mastocytosis with sunitinib
Source: F1000Res. 2017 Dec 28;6:2182. [Version 1] doi: 10.12688/f1000research.13343.1 (PMC5946163; doi:10.12688/f1000research.13343.1)
Supplement: Supplementary file 2 [file f1000research-6-14480-s0001.tgz › 327aef47-dccc-40a8-9b45-a1b27d3fc0a3.pdf]

Supplemental Table S2: C-Findings = Indication of impaired organ function due to MC infiltration defining a SM as an aggressive SM

Cytopenia(s): absolute neutrophil count < 1000/µL or hemoglobin < 10 g/dL or thrombocytes < 100,000/µL
Hepatomegaly with ascites and impaired liver function
Palpable splenomegaly with hypersplenism
Malabsorption with hypoalbuminemia and weight loss
Skeletal lesions: osteolyses or/and severe osteoporosis causing pathologic fractures
Life-threatening organopathy in other organ systems that is definitively caused by an infiltration of the tissue by neoplastic MCs

Based on e.g., Valent P. Systemic mastocytosis. Cancer Treat Res 2008;142:399-419.
