# Supplementary material for: Case Report: Treatment of systemic mastocytosis with sunitinib
Source: F1000Res. 2017 Dec 28;6:2182. [Version 1] doi: 10.12688/f1000research.13343.1 (PMC5946163; doi:10.12688/f1000research.13343.1)
Supplement: Supplementary file 3 [file f1000research-6-14480-s0002.tgz › bad1e909-12cb-4856-9a36-636cd4696dd3.pdf]

Supplemental Table S3: WHO 2008 Diagnostic Criteria for Systemic Mastocytosis (SM).[1]
WHO: World Health Organization; MC: mast cell.

Major Criterion:

1. Multifocal dense infiltrates of MCs (>15 mast cells in aggregates) in bone marrow biopsies and/or in sections of other extracutaneous organ(s) (CD117-, tryptase- and CD25-stained)

Minor Criteria:

1. Abnormal spindle-shaped morphology in >25% of MCs in marrow or other extracutaneous organ(s)
2. Abnormal marrow MC expression of CD2 and/or CD25 (i.e., co-expression of CD117/CD25 or CD117/CD2)
3. Activating KIT mutation at codon 816 in in marrow, blood, or extracutaneous organ(s)
4. Serum total tryptase > 20 ng/mL (does not apply in patients who have associated hematologic non-mast-cell lineage disease)

Diagnosis of SM made by either (1) major criterion + any one or more minor criteria, or (2) any three minor criteria.

1. Based on e.g., Horny HP, Metcalfe DD, Bennett J, Bain BJ, Akin C, Escribano L, Valent P. Mastocytosis. In: Swerdlow SH, Campo E, Harris NL, Jaffe ES, Pileri SA, Stein H, Thiele J, Vardiman JW.WHO Classification of Tumors of Hematopoietic and Lymphoid Tissues (4th ed.).Lyon, France: International Agency for Research and Cancer, 2008:54–63.
