# Supplementary material for: Case Report: Treatment of systemic mastocytosis with sunitinib
Source: F1000Res. 2017 Dec 28;6:2182. [Version 1] doi: 10.12688/f1000research.13343.1 (PMC5946163; doi:10.12688/f1000research.13343.1)
Supplement: Peer review history

# Peer review: Referee response for version 1

**DOI:** 10.5256/f1000research.14480.r29334

**Published:** 2018-05-14

**Authors:** Vicki Ratner

**Affiliations:** 1 Founder and President Emeritus, Interstitial Cystitis Association of America (ICA), McLean, VA, USA

First, I would like to clarify that I am a clinician, and not a researcher.  I have an expertise in mast cells, in particular, mast cells and interstitial cystitis. 

 I believe that it is very important to publish case reports, particularly on a disease that is relatively rare, and little is known about.  I applaud the authors in their efforts.  This case report focuses on a patient with aggressive systemic mastocytosis (ASM).  The drug used for treatment  was subitinib, which has been approved by the FDA for the treatment of renal cell carcinoma and imitinib–resistant gastrointestinal stromal tumors. 

 Sunitinib, an oral medication, is a multitargeted receptor tyrosine kinase (RTK) inhibitor, as opposed to imitinib, which is also an RTK inhibitor, but can only target one type of receptor.  20% of patients do not respond to imitinib, and 50% become resistant to this drug over time.

 The most common side-effects of sunitinib include HTN, fatigue, stomatitis, neutropenia and thrombocytopenia.  Other adverse events include weakness, diarrhea, nausea, anorexia, erythrocytosis (an increase in the mass of the RBC’s) and yellow discoloration of the skin.  In addition, the patient may develop hand-foot disease, also called acral erythema, where the palms of the hands and the soles of the feet become red, swollen, numb and cause the skin to peel off. Side-effects in patients using sunitinib occurred in 10% or less of cases, with the exception of HTN, which occurred with much higher frequency.

 There has been only one previous successful case report using sunitinib for aggressive systemic mastocytosis in the European literature.  Molderings, et al., reported that the patient experienced less fatigue, which made daily ADL’s (activities of daily living) easier for the patient to perform.  In addition, after treatment, the subcutaneous fibrous nodules became movable (as opposed to firmly attached to the underlying tissue) and the smaller nodules disappeared completely.  It is unclear why the remaining nodules, now movable, became tender.  

 In this second case report, where sunitinib was also used to treat progressive aggressive systemic mastocytosis, there were some benefits. The authors pointed out that the patient had complete resolution of his intense bleeding gums within just 3 days of treatment, and also reported less fatigue.  The subcutaneous nodules also became movable and tender.  However, the patients’ body hair became white, and there was a decrease in the number of thrombocytes and total protein in the blood.  The uric acid level increased to the point of causing gout. At this point, the authors agreed that the side effects outweighed the benefits, and the medication was discontinued.

   There is one case report published by L. Afrin in 2015 about the use of sunitinib in a patient with Mast Cell Activation Syndrome (MCAS) here in the U.S.  The patients’ symptoms have completely resolved as long as she remains on the drug, and thus far she has had no side effects.  It has been over a year now.

 The cost of sunitinib (Pfizer) is extremely high - almost $150,000 per year.  Often, in the U.S., insurance companies have refused to pay for all or part of the costs of this drug, so the cost to the patient can be a tremendous burden.  Sunitinib most likely costs less if the dose is lowered and used cyclically.  Cyclical use (for example 4 weeks on followed by 2 weeks off) may actually decrease the side effects more than a lower daily dose.  However, though cyclical use may be sufficient to control malignancy, it is unlikely that cyclical use would be sufficient to control dysfunctional mast cells, as in MCAS, which might quickly revert to a more symptomatic baseline state of inappropriate activation as soon as the plasma concentration of the drug drops below a therapeutic threshold.

I have read this submission. I believe that I have an appropriate level of expertise to confirm that it is of an acceptable scientific standard.

# Peer review: Referee response for version 1

**DOI:** 10.5256/f1000research.14480.r33390

**Published:** 2018-05-10

**Authors:** Leonard Weinstock

**Affiliations:** 1 Washington University School of Medicine, St. Louis, MO, USA

*Is the background of the case’s history and progression described in sufficient detail?*

 The background in the Introduction section nicely details the challenges treating systemic mastocytosis. This disease has considerable morbidity based on mediator secretion by malignant mast cells. The discussion clarifies why reduction of mast cell burden by other drugs do not necessarily bring about symptomatic relief.

*Are enough details provided of any physical examination and diagnostic tests, treatment given and outcomes?*

 The case report second along with the tables provided excellent details that enable the reader to understand the scope of systemic mastocytosis in this subject, the severity of symptoms, and the challenges in treatment. The patient failed standard therapy for this disease and yet had significant relief with a new medical approach. The problematic side effect of gout might have been prevented by use of allopurinol.

*Is sufficient discussion included of the importance of the findings and their relevance to future understanding of disease processes, diagnosis or treatment?*

 Yes. Further studies are warranted.

*Is the case presented with sufficient detail to be useful for other practitioners?*

 Yes. There is excellent detail to understand the management of systemic mastocytosis.

I have read this submission. I believe that I have an appropriate level of expertise to confirm that it is of an acceptable scientific standard.

# Peer review: Referee response for version 1

**DOI:** 10.5256/f1000research.14480.r29333

**Published:** 2018-01-08

**Authors:** Michael Huber

**Affiliations:** 1 Institute of Biochemistry and Molecular Immunology, Medical Faculty, RWTH Aachen University, Aachen, Germany

Systemic mastocytosis as one form of systemic mast cell activation disease usually is an incurable disease, which due to patient-specific genetic and/or epigenetic alterations requires a highly personalized therapy to mitigate consequences of mast cell proliferation and/or inflammatory activation. This also implies that a few patients might profit from a therapy, which usually is referred to as noneffective for the majority of patients. Therefore, for the practitioner the publication of such rare cases is important to allow for a potentially effective, though unusual therapy option.

 The present case report by Molderings *et al.* offers a detailed description of a patient with aggressive systemic mastocytosis treated with the tyrosine kinase inhibitor (TKI) sunitinib as well as an objective statement about pros and cons of the treatment. Moreover, this case report offers important background knowledge on the current classification of primary mastocytosis (Suppl. Table S1), a list of C-findings = indications of impaired organ function due to mast cell infiltration defining a systemic mastocytosis as an aggressive systemic mastocytosis (Suppl. Table S2), and the WHO 2008 diagnostic criteria for systemic mastocytosis (Sull. Table S3). These enable the reader to access this information important for the comprehension of this case report without obtaining the original literature.

 The reviewer suggests the following changes/additions:       

 a) In the description of the TKI sunitinib, the publication by Todd *et al* (Todd JR, Becker TM, Kefford RF, Rizos H. Secondary c-Kit mutations confer acquired resistance to RTK inhibitors in c-Kit mutant melanoma cells. Pigment Cell Melanoma Res. 2013; 26: 518-526) should be added. It describes a melanoma cell subline with an additional T670I mutation in c-Kit, which shows resistance to the TKIs imatinib, nilotinib, and dasatinib, but responds to sunitinib.

 b) In Suppl. Table S3 (minor Criteria), the sentence “3. Activating KIT mutation at codon 816 in in marrow, blood, or extracutaneous organ(s)“ should be changed to “3. Activating KIT mutation at codon 816 in marrow, blood, or extracutaneous organ(s)“

I have read this submission. I believe that I have an appropriate level of expertise to confirm that it is of an acceptable scientific standard.
